# Scientist and data architect collaborate to curate and archive an inner ear electrophysiology data collection

**DOI:** 10.1371/journal.pone.0223984

**Published:** 2019-10-18

**Authors:** Brenda Farrell, Jason Bengtson

**Affiliations:** 1 Bobby R Alford Department of Otolaryngology and Head & Neck Surgery, Baylor College of Medicine, Houston, Texas, United States of America; 2 K-State Libraries, Kansas State University, Manhattan, Kansas, United States of America; King Abdullah University of Science and Technology, SAUDI ARABIA

## Abstract

In the past scientists reported summaries of their findings; they did not provide their original data collections. Many stakeholders (e.g., funding agencies) are now requesting that such data be made publicly available. This mandate is being adopted to facilitate further discovery, and to mitigate waste and deficits in the research process. At the same time, the necessary infrastructure for data curation (e.g., repositories) has been evolving. The current target is to make research products FAIR (Findable, Accessible, Interoperable, Reusable), resulting in data that are curated and archived to be both human and machine compatible. However, most scientists have little training in data curation. Specifically, they are ill-equipped to annotate their data collections at a level that facilitates discoverability, aggregation, and broad reuse in a context separate from their creation or sub-field. To circumvent these deficits data architects may collaborate with scientists to transform and curate data. This paper’s example of a data collection describes the electrical properties of outer hair cells isolated from the mammalian cochlea. The data is expressed with a variant of The Ontology for Biomedical Investigations (OBI), mirrored to provide the metadata and nested data architecture used within the Hierarchical Data Format version 5 **(**HDF5) format. Each digital specimen is displayed in a tree configuration (like directories in a computer) and consists of six main branches based on the ontology classes. The data collections, scripts, and ontological OWL file (OBI based Inner Ear Electrophysiology (OBI_IEE)) are deposited in three repositories. We discuss the impediments to producing such data collections for public use, and the tools and processes required for effective implementation. This work illustrates the impact that small collaborations can have on the curation of our publicly-funded collections, and is particularly salient for fields where data is sparse, throughput is low, and sacrifice of animals is required for discovery.

## Introduction

Traditionally, scientists publish reports that describe their experimental findings, while data collected to uncover these findings are not typically reported, nor are they made readily available to their scientific peers, despite their value for further discovery and public education and awareness. There are notable exceptions where it was deemed beneficial to the disciplines and in the public interest that scientific data collections be aggregated, stored as collections, and made generally available. They include: X-ray crystallography of chemical structures, [e.g., Protein Data Bank] [1], with the caveat that the raw diffraction data is only now being archived with coordinates [2]; sequencing of genes [NCBI] [3]; characterization of astronomical objects and their radiation (e.g., CADC and NASA’s HEASARC) [4, 5]; and weather-associated measurements (e.g., NOAA national centers for environmental information) [6]. There has been a growing chorus of voices [7–12] arguing that such practices should be extended to other types of data because it permits checking the data and analysis; it allows for aggregation of data thereby improving the robustness of findings by increasing sample size, and it facilitates re-use of data. All of these factors should reduce unnecessary and costly experiments while helping to improve the reproducibility of the methods, results and inferred findings within biomedical sciences [13]. This is particularly relevant now when the lack of reproducibility is a well-documented deficiency that results in an enormous cost to the taxpayer [14].

The need to share and aggregate data is even more obvious in fields where the experimental throughput is notoriously slow and where it is usually necessary to sacrifice experimental animals in the interest of scientific discovery. This is the case within cochlear physiology. Measurements require specialized equipment for the detection of movements [15, 16], pressures [17], potentials [18–20], or currents [21–23] that reflect the physiological responses of mammals to acoustic stimulation. Such assays also require healthy mammals (e.g., gerbils, guinea pigs) that can survive the surgeries and that exhibit minimal run-down upon perturbation with probes and sensors. These procedures are made more difficult because experimental access to the cochlea is limited by the bony labyrinth which is the most petrous part of the temporal bone. Typically results of sophisticated measurements demand the sacrifice of many animals over many years [19]. Similar problems arise when studying the electrical and mechanical properties of the sensory epithelium *ex vivo*. The sensory hair cells (namely the outer hair cells and inner hair cells) lose their viability quickly upon interrogation, and the electrophysiological techniques (i.e., voltage clamp or current clamp) necessitate specialized training and equipment. In addition, the properties (i.e., morphological, electrical and mechanical) of the hair cells can differ because of the tonotopic architecture of the cochlea; a topographic map that relates the characteristic frequency to place. Hair cells located in the base of the cochlea respond to higher frequencies (up to 43 kHz in the guinea pig) with the best frequency decreasing towards the apex of the cochlea, reaching a minimum at the apex (e.g., the guinea pig is at 0.060 kHz) [24]. We and others have used these variations to describe tonotopic relationships that relate the properties (i.e., physical [25–27], electrical [28–31], or expression levels of proteins [32, 33]) with the place (or best frequency) within the cochlea. Given the number of hair cells within a cochlea, it is a formidable task to record or define these properties along the entire tonotopic axis, and this provides further motivation for aggregation of data collections in auditory physiology. Pioneering efforts in other sub-fields show that aggregation of electrophysiological data for peer and public re-use is completely feasible [34]. For example, Eglen and colleagues combined twelve published data collections of time series data of action potentials of the retina, formatted the data collections to the same standard, and made comparisons across them. The number of recordings produced by sharing data is 366 compared to 30 recordings produced by each laboratory without data sharing. The algorithms used to analyze the data were provided on-line for readers to reproduce the results. By combining data collections, the sample size and hence the power is enhanced, while sharing the algorithms provides the reader a means to verify the results. This improves data robustness and should reduce the number of animals required to make new findings [35].

Despite these logical arguments, the electrophysiological community is slow to adopt practices to share electrophysiological data collected from the hearing (e.g., mammalian cochlea) or balance (e.g., semicircular canals, utricle, and saccule) organs. Efforts have been made for quite some time to preserve tissue especially human temporal bones, and register these specimens with the National Temporal Bone Database [36]. This database contains data and metadata (e.g., age and sex of specimens) associated with about 8000 specimens from 23 US hospitals and laboratories. More recently, audiological data relevant to human pediatric hearing health can be found and deposited through the AudGenDB portal [37]. This is a web-based query database (beta version, v2.0) with clinical data (e.g., audiograms, tympanograms, computerized tomography scans) from about 96,000 pediatric patients with plans to add genetic information in updated versions of the portal. A more recent initiative is a web portal that enables the sharing of gene expression data within both the auditory and vestibular systems across species via the gEAR (gene Expression Analysis Resource) portal [38]. The International Mouse Phenotyping Consortium, IMPC [39] is characterizing the genotype and phenotypes of mice with a pipeline that includes performing hearing tests with auditory brain stem methodology [40]. This resource allows peers and the public to download the data produced by the consortium, but it does not provide a place for others to deposit and share their data.

Effective data sharing is not a trivial undertaking. It requires new infrastructure, adoption of new practices, and consensus by the investigators within disciplines. The impediments that still exist include: (i) the desire by some scientists to keep their data confidential for their own latent discoveries; (ii) the need to assimilate data, including relevant descriptions or metadata, into one package or place (which can be time consuming), such as collating hand-written laboratory notes with data stored on PCs and servers [41]; and (iii) proper curation of data collections requires training within the field of information and information systems which most scientists do not possess. The necessity to devise a standard process with rules to cite, find and consistently access a data collection was articulated by Altman and King [42]. A detailed list of encompassing basic principles was developed by a diverse group of people with leadership from FORCE11 (aka Future of Research Communication and e-Scholarship [43]). This group drafted and published the FAIR principles which stipulate that all digital objects (including data collections) should be Findable, Accessible, Interoperable and Reusable where these adjectives are applied to both humans that make use of them and machines that survey them [12]. These principles are part of a living document [43] that all stakeholders (e.g., researchers, data architects, journals, publishers, and repositories) should strive to adopt to facilitate good stewardship for digital objects including data collections. A recent report describes a roadmap to hasten citation of data collections [44].

To hasten discovery in auditory electrophysiology and to facilitate effective data sharing (addressing impediment (iii)), a scientist, (BF) initiated a collaboration with a data architect (JB) to transform electrophysiological data from private to public use. This data collection describes the electrical properties of outer hair cells isolated from the mammalian cochlea of guinea pigs. At the onset, it was paramount that the information specialist, and not the scientist, provide the rationale for the data design. This ensured that the data collection was annotated with expansive metadata (cf. [41]). This was achieved by describing the data with a purpose-built variant of the Ontology for Biomedical Investigations [45]. The data, originally stored in the proprietary MATLAB [46] format, was then re-arranged and translated to Hierarchical Data Format version 5 **(**HDF5), [47] a non-proprietary format, using a group and attribute structure based upon this variant OBI ontology. Early and condensed version of this work was presented at the 2018 International Conference on Biological Ontology (ICBO) and subsequently published online [48].

We describe our data management plan and how it was implemented to produce a data structure that starts to meet the FAIR principles [12] and demonstrate that some of the barriers to data sharing (item iii) can be mitigated by undertaking a two-way collaboration. This illustrates a point made by others: that buzzwords like *Big Data* can be a misnomer. Impactful data does not always entail hundreds of users or terabytes (TBs) of data; it can also refer to the potential positive impact that small collaborations [49] have on the production, curation, and sharing of our publicly-funded collections.

## Description of data

This data collection describes the linear and non-linear electrical properties of the outer hair cells of the domestic guinea pig. The data is generated by whole-cell voltage clamping isolated outer hair cells and determining the linear capacitance and voltage-dependent membrane capacitance. This technique was developed over 30 years ago [50] with much of the methodology refined for outer hair cells in the ensuing years. This method is commonly used to establish whether cells isolated from wildtype or engineered rodents exhibit the characteristic voltage-dependent capacitance in response to a change in the membrane potential. Typically, a cell is whole-cell voltage-clamped and electrical admittance monitored during a DC voltage ramp. In our experiments, the admittance was interrogated with a two-sine stimulus and the membrane capacitance calculated at each potential from this admittance [28, 51, 52]. The membrane resistance and access or series resistance of the pipette was also calculated. A computer program was written in LABVIEW for Windows (v8.5.1) in conjunction with a digital to analog converter card (PCI-6052E, National Instruments Austin, TX) that controlled the calibration, stimulus, and acquisition of the admittance. The data was exported as a spreadsheet into Microsoft Excel (Office version 2003 and later versions), and then later imported and analyzed in MATLAB (v. 8.2-v 9.0) [46]. In some cells, we also measured the DC conductance by interrogation of the cell with a voltage-step function which was calculated from the change in the mean steady-state current with respect to DC voltage.

The electrophysiology data associated with each recording was assimilated for each recording from an outer hair cell and saved as an *array of structures* in MATLAB where the field-name is common across all cells, and the value associated with the field name can be retrieved by MATLAB syntax. Each recording of an outer hair cell has 82 fields. The field names used were originally chosen by the scientist for manipulation within the MATLAB environment and do not reflect a class or sub-class of an ontology.

## The rationale for data design

The value of a research data collection is intrinsically magnified by two factors: aggregation potential and future scalability. Data that can be effectively aggregated may be integrated into systematic reviews or meta-analyses to magnify their value. Data that retain their interpretive value over time may continue to be used far into the future, even as the context the data originally existed within changes, allowing them to scale into the future effectively. At the other extreme are situations when data or information objects are not described with a sufficient degree of accuracy, or are described without *context*, potentially leading to *orphaned data* or objects that are useless outside of their original context, be that context a particular laboratory, the guiding elucidation of a specific researcher, or a time and place in which particular jargon or conventions are used [53, 54]. To successfully implement a robust description (i.e., Knowledge Representation (KR) [55]) of the data, our strategy is to use expansive metadata to describe the data structure. This provides essential context to the data so that others who reuse it are not forced to attempt to re-create or guess at that context themselves. This approach allows researchers to avoid relying on commonly understood (and potentially misunderstood) jargon, and it mitigates the need to contact the original owner of the data. Such a preserved context provides the researcher with the assurance that the data represents exactly what the owner intended.

To preserve the original data context and meaning, and allow it to “take its place within our general understanding of the world” [56], such contextualization should allow for human understanding of the data, both across disciplines and in the same discipline across time [57]. It should also allow data collections to be interoperable so that they can be aggregated together to form a more complete picture of the subject being researched. Such interoperability can result from the authoring of *ad hoc* translation programs [58]. In some cases, efforts to aggregate data have been forced to deal with unstructured data (such as data in simple tables, spreadsheets, or text files), greatly increasing the time needed to edit and reuse the data in question [59]. However, by creating more structured data (e.g., eXtensible Markup Language (XML), JavaScript Object Notation (JSON), HDF5, and relational database models) which facilitate navigation and search of values, and making use of metadata to capture the original data context in a portable way, data interoperability can be achieved with much less effort [58].

To organize the data collection appropriately with metadata, the metadata itself must have structure, so that terms and definitions employed have unambiguous meanings that scale across time to provide an accurate classification of the generated data collections [60]. Metadata should be structured logically to provide a framework to connect the data within a data collection to the rest of the world in a meaningful way. One tried and true modality for achieving this is to employ an ontology [61]. Ontologies are information structures that contain formal terms with definitions (generally describing a particular discipline or technical area) and descriptions of the relationships of those defined terms to one another. Technically, ontologies are differentiated from simpler classification schemes because they allow sub-classes to fall beneath more than one parent class [56]. Ontologies not only provide a rich contextual environment for data but they also, when that ontology is used as a framework for the data values, potentially make interoperability easier by providing a data collection with a predictable and logical format to traverse the data file with a computer program. Data described by an ontology elucidates meaningful relationships among the concepts described by that data. Such a depth of knowledge can only be derived from less structured data through data mining by computationally challenging techniques [62]. Many scientists are unaware of the importance of metadata and ontologies to the preservation of data. The developers of the CARMEN (code analysis, repository, and modeling for E-neuroscience) portal [63] described the problems they encountered when trying to encourage scientists to use expansive descriptions for their uploaded data, forcing them to resign to the use of minimal descriptions. As a result, they were not able to produce an ontology-driven metadata system and cautioned that such portals can become "data-dumps", where effective sharing is difficult without sufficiently descriptive metadata [41].

In addition to the need for ontologies, consensus on other best practices to implement effective sharing of electrophysiological data has received attention by consortia (e.g., International Neuroinformatics Coordinating Facility INCF [64]; Neuroscience Information Framework (NIF) [65]; Neurodata Without Borders (NWB) [66]; and the CARMEN consortium [63]). Most of the focus evidenced by these groups is on organizing and sharing imaging data, as demonstrated by the successful development of XNAT [67]. The minimum information that should be reported about an (electrophysiology) neuroscience investigation (MINI) was published by the CARMEN [68] and by Collaborative Research in Computational Neuroscience (CRCNS) consortia [69–71]. Cardiac electrophysiologists have proposed minimum standards for reporting results of cardiac electrophysiology experiments (MICEE) [72] and developed several consortia to share data; e.g., Experimental Data and Geometric Analysis Repository, EDGAR [73] and Consortium for ECG Imaging [74]. To improve outcomes for epilepsy and seizures similar efforts have been done within this community [75–77]. There have also been efforts to develop a standard format to store data [70] to facilitate easier integration of disparate data collections. In 2014 the electrophysiological taskforce of the INCF [78] proposed that the standard format for electrophysiological data storage should be based upon Hierarchical Data Format version 5, (HDF5) [47]. This non-proprietary format was developed by the HDF group for long term storage of large or complex data collections and is compatible with common operating systems (i.e., Linux, Unix, Mac, and Windows). It provides significant flexibility and integration with a variety of application programming interfaces, including C, MATLAB, FORTRAN, Java and Python.

Other file formats include XML and JSON. XML provides a flexible data format, albeit one which is entirely hierarchal in nature. The Web Ontology Language (OWL) files commonly used to store ontologies are, in fact, simply a subset of XML. However, this flexibility comes at a significant cost, since formatting complex data, such as those found in this data collection, would require an extraordinary amount of markup. XML is a markup language designed to encode text characters through the use of “tags” (similar to HTML). It has no built-in complex data types or objects. Data existing as matrices or arrays as commonly found in electrophysiology or biophysical data collections would have necessitated the creation of a very complex Document Type Definition (DTD) for the file, and the programmatic application of an unwieldy amount of markup which would not have been easy for other researchers to navigate when they opened the file in a text or XML editor.

Similarly, while JSON is a very flexible, object-based data transfer and storage format, it lacks the built-in data types that are such an asset of HDF5. MATLAB functions exist for the conversion of MATLAB data to XML or JSON, however, given that both of those formats are primarily designed for the encoding of text, they were deemed to be inadequate. The combination of a hierarchal format, with embedded, complex data types, and the ability to easily associate those data with rich, descriptive metadata both implicitly (through the hierarchal structure), and explicitly (through the use of attributes), made HDF5 a clearly superior choice. In addition, HDF already enjoyed significant adoption by scientists, making it likely that it would scale into the future effectively, and that sufficient means would exist to migrate the data collection to another file format from HDF5 if necessary. The eponymous hierarchal format of HDF5 makes it extremely versatile, allowing for a multiplicity of data architectures and strategies to arrange and describe the data.

## Data management plan

### Develop an ontology for data description

The ontology we used is a variant and extension of the Ontology for Biomedical Investigations (OBI) [45] that was developed to describe the diverse range of assays used in biological and bioengineering-based research and discovery. We used OBI because it already contained many of the classes and relationships that we needed, and it was practical to build upon this effort. When adding classes to the existing OBI ontology we followed the general guidelines of the Minimum Information to Reference an External Ontology Term (MIREOT), a standard encouraged by the architects of OBI, who also stipulate that existing ontologies be used and modified where needed instead of creating new ones [45, 79]. We made use of several bioportals and tools: the National Center for Biomedical Ontology, (NCBO) bioportal [80]; the Ontology Lookup Service [81]; Ontobee [82, 83]; and OntoMaton [84] to locate classes and their definitions to describe the data. Classes from ontologies were grafted into OBI, along with custom classes added for this data collection; employing a technique known as Application Profiles to produce a variant ontology [57] named *OBI based Inner Ear Electrophysiology (*OBI_IEE*)*. We use this notation in compliance with another application ontology also built upon a version of OBI that describes Beta Cell Genomics Ontology (OBI_BCGO) [85]. When editing the ontology we used the Protégé editor [86], while regularly deploying newly edited versions of the ontology to the web-based version of the tool, WebProtege [87]. To follow the relationships within the data the OWL file can be downloaded from the repositories holding the data collection, and from the NCBO bioportal and perused with Protégé editor.

**Fig 1:**
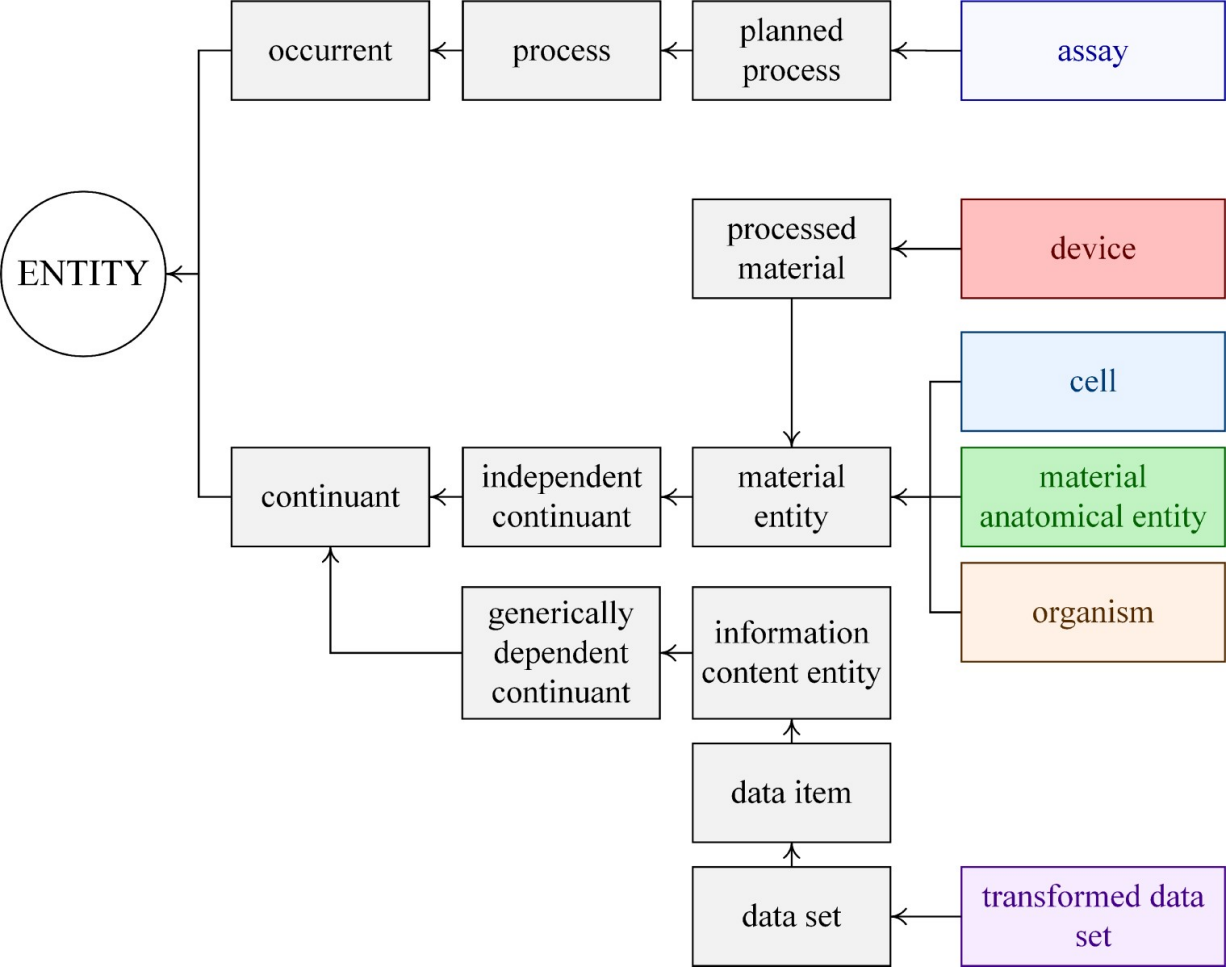
Six (6) classes that describe the data. The classes are denoted with different colors. Arrow denotes *is-a* property.

The data collection is described by six main branches and shown in Fig 1. This class structure was derived through a logical mapping of OBI’s existing classes onto the experimental data. The branch that describes the electrical recordings (i.e., voltage-clamp measurements) is the *assay*. *Device* constitutes the 2^nd^ branch as instruments (e.g., amplifier) are required to perform this assay. The voltage-clamp measurements were performed on cells, hence *cell* was chosen as a 3^rd^ branch. The cells were isolated from the cochlea of guinea pigs, therefore *anatomical entity* and *organism* are natural 4^th^ and 5^th^ branches. We introduced the new class *transformed data set* to describe the 6^th^ branch. It describes the data sets produced upon the use of one or more data transformation processes. For example, it includes partitioning the measurement data into the voltage-independent and voltage-dependent data sets that are typical of this data. These branches and the constituent classes of which they are composed represent the main nodes within the data collection. They (along with the metadata) describe the experiment (including the animals used), the general properties of the experimental animals, information about the devices used for the assay, and information about the type of assay performed.

**Fig 2:**
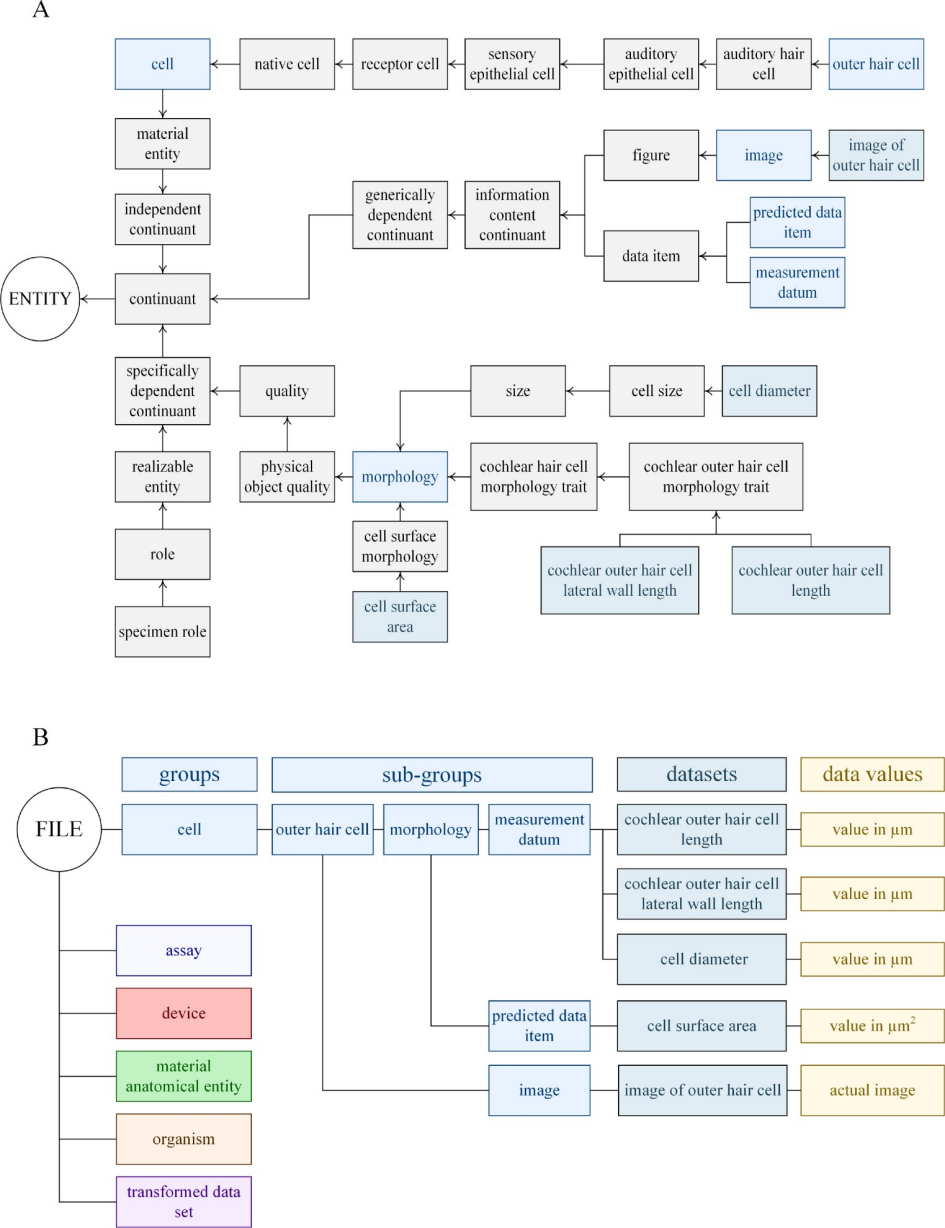
Directed root tree and data architecture of the *cell* arm of the data. (A) The classes that describe the *cell* arm data. For clarity, we do not show that *cell surface area* was a predicted data item and that *cell diameter*, *cochlear outer hair cell lateral wall length* and *cochlear outer hair cell length* were measured data items. (B) The groups that describe *cell* arm with other main groups. The classes that were transformed into sub-groups and datasets are denoted by blue and aqua.

We provide the directed root tree for each branch and the number of new classes and the number of terms imported from each ontology in Table 1. Consider the branch *cell* (Fig 2); the experiments were performed with outer hair cells which we mapped to this sub-class by use of classes derived from the Cell Ontology (CL) [88]. The morphological characteristics, including the size of each *outer hair cell* used in an experiment, were measured from an *image* obtained by an *analog camera* during the experiment and include *cochlear outer hair cell length* and *diameter* of the outer hair cell. These experimental measures are described with classes (e.g., *cell diameter*) derived from the Ontology of Biological Attributes (OBA) [89]. The *cell surface area* class was predicted from these measurements and the description imported from OBA. In this case, we delineate whether a *data item* was measured or predicted as this enhances the understanding of the methodology. The main classes of *morphology* and *size* were imported from Phenotype and Trait Ontology, PATO [90]. We also measured the *cochlear lateral wall length* which we introduce as a new class, and make it a sibling of *cochlear outer hair cell length*. We described the *organism* arm in our short report [48]. The *anatomical* arm (**S1 Fig**) made use of the anatomical structure terms imported from the Foundational Model of Anatomy (FMA) [91], and Uberon multi-species anatomy (UBERON) [92, 93] ontologies. To describe the positional origin of the cells interrogated we imported terms from PATO, and define three further new classes that describe their position: *cochlear turn*, *apical*, and *basal*. These terms are commonly used by auditory scientists.

**Table 1:** Ontologies and number of classes imported and created for *OBI based Inner Ear Electrophysiology (*OBI_IEE*)* variant.

Ontology	International Resource Identifier	Imported classes	Reference
CHEBI	http://purl.obolibrary.org/obo/chebi.owl	8	[94]
CL	http://purl.obolibrary.org/obo/cl.owl	5	[88]
CNO	https://bioportal.bioontology.org/ontologies/CNO.owl	2	[95]
EDAM	http://edamontology.org/EDAM.owl	2	[96]
FMA	http://purl.obolibrary.org/obo/fma.owl	12	[91]
GO	http://purl.obolibrary.org/obo/go.owl	6	[97]
MP	http://purl.obolibrary.org/obo/mp.owl	5	[98]
NCBITaxon	http://purl.obolibrary.org/obo/ncbitaxon.owl	2	[99]
NCIT	http://purl.obolibrary.org/obo/ncit.owl	2	[100]
OBA	http://purl.obolibrary.org/obo/oba.owl	7	[101]
OPB	https://bioportal.bioontology.org/ontologies/OPB.owl	1	[102]
PATO	http://purl.obolibrary.org/obo/pato.owl	28	[90]
SBO	http://purl.obolibrary.org/obo/sbo.owl	1	[103]
SIO	http://semanticscience.org/ontology/sio.owl	3	[104]
UBERON	http://purl.obolibrary.org/obo/uberon.owl	2	[93]
*New*		37	
Total Classes		123	

**Fig 3:**
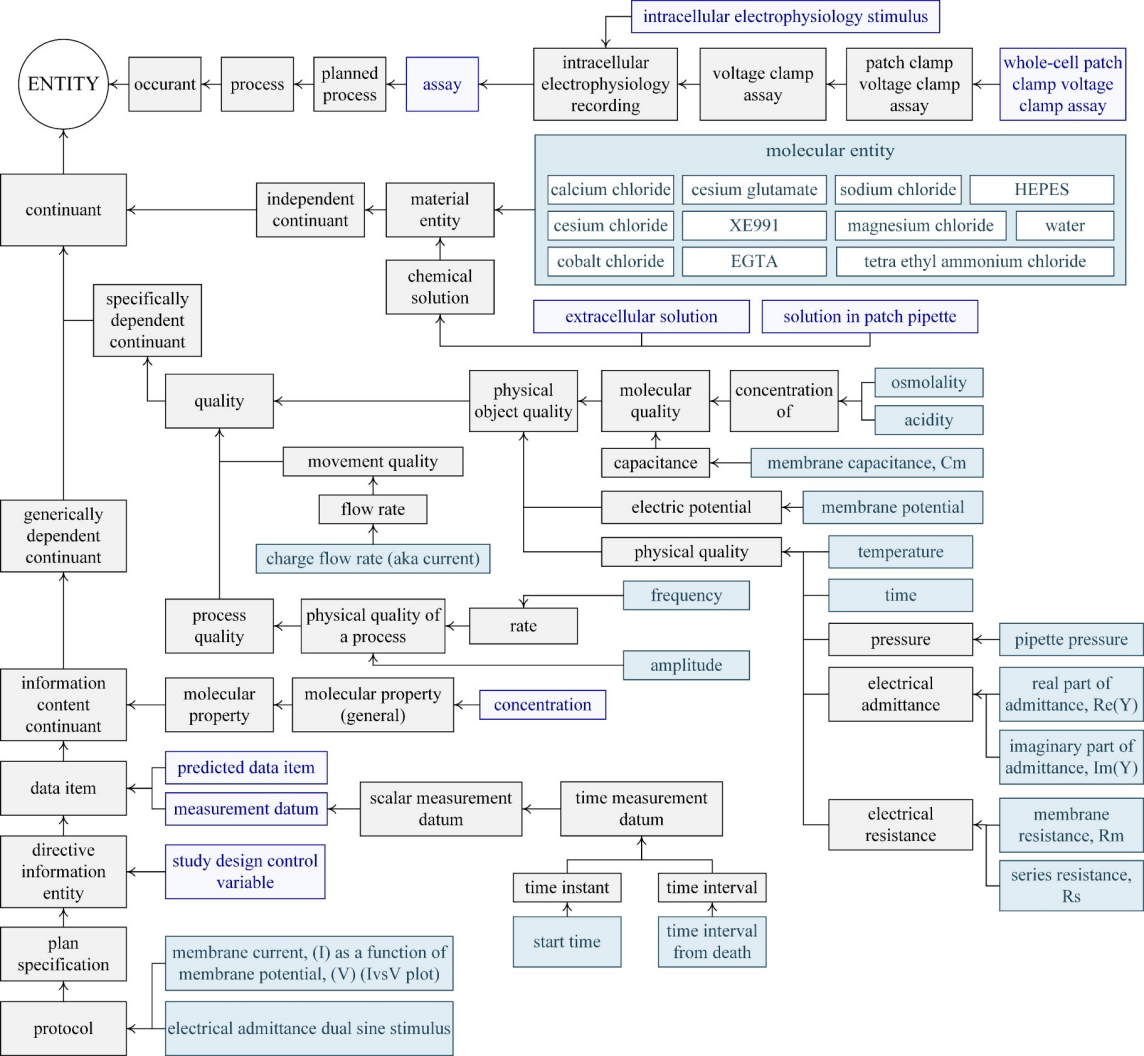
Directed root tree for the *assay* arm showing the classes that describe the data for both protocols. For clarity, we do not show that *membrane capacitance*, *membrane resistance*, and *series resistance* were *predicted data items* and the *electrical admittance* and *charge flow rate* (aka current) were *measurement data items*. The classes transformed into sub-groups and datasets are denoted by purple and aqua.

The directed root tree of the *assay* arm, which is more elaborate than the other branches, is shown in **Fig 3.** This is a planned process where the cells were interrogated by voltage clamping with a *whole-cell patch-clamp voltage clamp assay* with two *protocols*. The main *protocol* was the *measurement of the electrical admittance with dual-sine stimulus*. To describe the stimulus used to interrogate the cells for each protocol, a common feature of such an assay, we introduce the new class *intracellular electrophysiology stimulus*. In this case, it describes the *frequency* and *amplitude* of the sine waves, and the magnitude of DC potential used to voltage clamp the *membrane potential* and the length of *time* this potential is held at this value. This assay measures the *real* and *imaginary* component of the *electrical admittance* which we introduce as new classes. The *membrane capacitance*, *membrane resistance*, and *series resistance* are calculated from the admittance based upon a model where we introduce the *series resistance* as a new class. Once again we delineate whether this *data item* is either predicted or measured. The time the assay commenced and this time relative to the life-death temporal boundary of the animal were both measured. In these experiments, the study design control variables are reported and include *temperature* and *pipette pressure*. We introduce *pipette pressure* as a new class, and we also define *study design control variable* as a class. In electrophysiology experiments, the solutions used affect the outcome, so for these, we introduce two new classes. The first is the chemical solutions used to bathe the cells (i.e., *extracellular solution*) and the second is the *solution in the patch pipette*. We import the classes for the compounds used to make up the chemical solutions from the Chemical Entities of Biological Interest (CHEBI) ontology [94], the class for their concentration from the EDAM ontology [96], and the classes describing the *acidity* and *osmolality* of the solutions from PATO.

The *device* arm is straightforward (S2 Fig) and defines three new classes that describe the *pipette pressure clamp*, *patch pipette*, and *analog camera*. The final arm includes the *transformed data set* (S3 Fig) which provides the data items normally provided in scientific reports. In this case, we define sixteen new classes including *linear membrane capacitance*, *non-linear membrane capacitance*, the *potential of maximum sensitivity* (often abbreviated to: *V*_0.5_, *V*_*peak*_ or *V*_*pk*_) and *sensitivity of a process to voltage* (often abbreviated by Greek symbol, α). Together, the branches (e.g., Figs 1–3) implicitly and explicitly containing this descriptive metadata provided a coherent framework we could hang the experimental data upon.

This project required importing 86 classes from fifteen [15] ontologies. The additional ontologies include Computational Neuroscience Ontology, (CNO) [95], Gene Ontology (GO) [97], Mammalian Phenotype Ontology, (MP) [98], Ontology of Physics for Biology, (OPB) [102], Semantic Science Integrated Ontology (SIO) [104], Systems Biology Ontology (SBO) [103], National Cancer Institute Thesaurus (NCIT) [100], and National Center for Biotechnology Information (NCBI) Organismal Classification (NCBITaxon) [99]. By mapping these data and metadata onto an application ontology: *OBI based Inner Ear Electrophysiology* (OBI_IEE), the logical connections of the data are preserved, which should enhance opportunities for search and discovery of this data [60]. By combining the data and metadata together, researchers seeking to reference and re-use the data should find sufficient qualitative context to make meaningful use of the data into the future [54].

### Design data architecture based upon ontology

Once the basic class structure was formulated (Fig 1), and the directed root trees compiled (e.g., Figs 2 and 3), it became obvious to both of us that such maps provide a framework that could be extracted to organize the data. We mirror the class structure established with the variant OBI ontology to arrange the data within Hierarchal Data Format version 5 [47]. The scientist with knowledge of the data and sub-field drove the choice of the classes that became part of the structured data collection.

The six *classes* of the ontology became the six main *groups* (group is a particular term that is a part of the HDF5 standard) within the HDF5 format, with abbreviated nomenclature when appropriate; i.e., *anatomical entity* was shortened to the group *anatomical*. The sub-classes within each class of the variant ontology were not translated; only sub-classes directly associated with the data collection (usually the adjacent sub-classes) became *sub-groups* within the translated data. For example, for the group *anatomical*, there are two sub-classes translated to a sub-group *subdivision of bony labyrinth* and *position*. In HDF5, data values are stored in datasets (dataset is a keyword in the HDF5 standard). In this case, the question of whether the *position* of the cell was found in the *apical* or *basal* regions of the cochlea was addressed by the dataset coined *apical-basal polarity*. Expanding downwards from the sub-group *subdivision of the bony labyrinth* provides the sub-group (the *subdivision of cochlea*) and the datasets (*cochlear turn and cochlea*) that describe whether the *left* or *right cochlea* was used and which *cochlear turn* was the origin of the cell (S1B Fig). In the same way, for the class *cell*, the first hierarchal sub-class translated to a sub-group is *outer hair cell* with *morphology* becoming a major sub-group with the datasets that are delineated based upon whether the data was *predicted* from a model or *measured* (Fig 2B).

In translating the class and sub-class structure of the ontology to HDF5, we embraced MINI guidelines [68, 71]. For example, the specimen was collected from a guinea pig and the characteristics of this organism (e.g., sex, phenotype) are described under the group *organism* [48].

**Fig 4:**
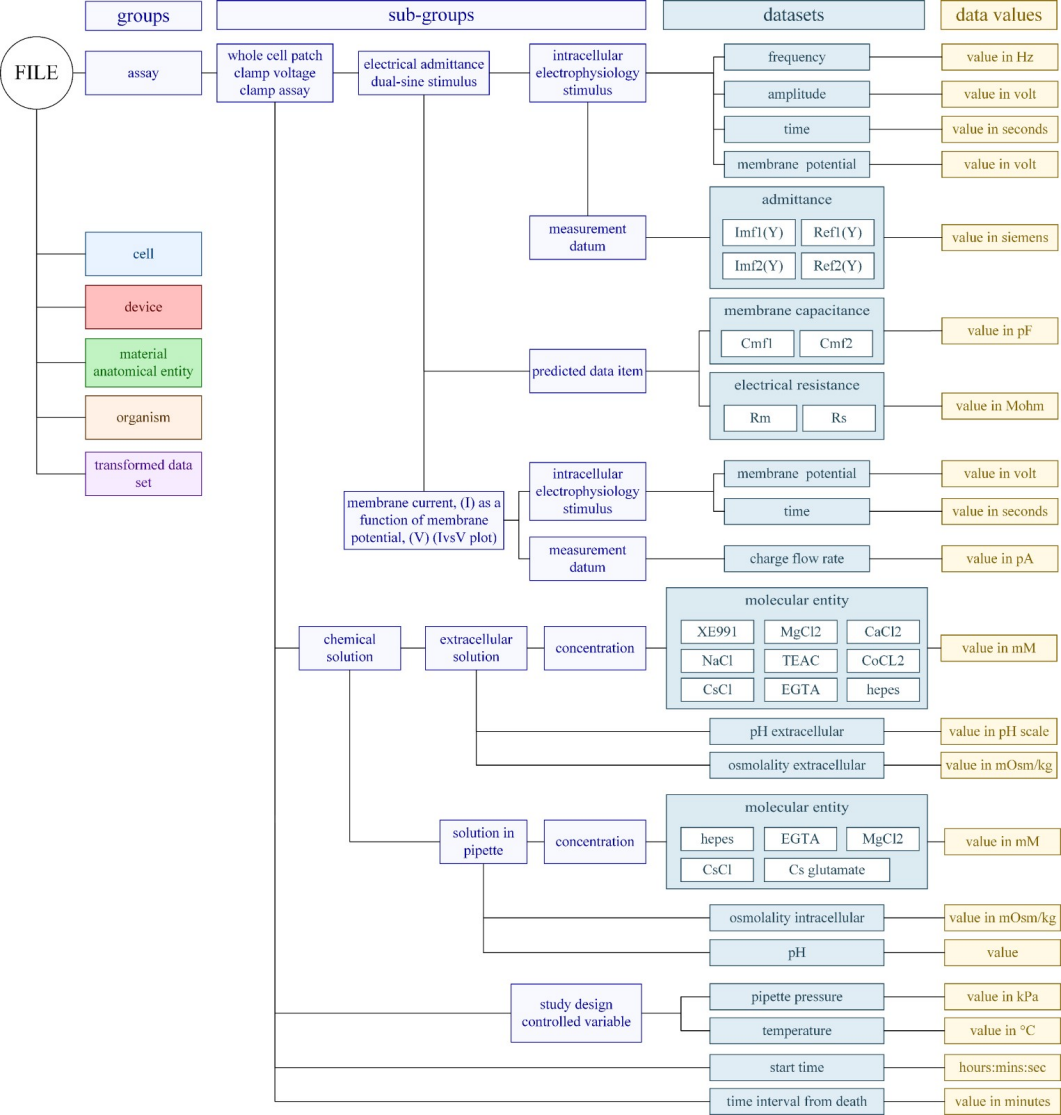
Data architecture for the two protocols of the *assay* arm of the data with other main groups shown. Refer to Fig 3 for the directed root tree for this arm.

The recording, experimental and stimulus conditions are classified under the group *assay* for each protocol (Fig 4). The sub-group *concentration* contains the ionic composition of the *extracellular solution* and the *solution in the patch pipette*, including their pH and osmolality, as required [68, 71] since electrophysiology results depend upon the ionic composition of the solutions. We added the chemical components of the solutions as labels of the dataset, with the molarity of each compound saved as the value within the dataset. We include the study controlled variables *pipette pressure* and *temperature* and we specify the *time* when the assay was conducted and the time interval between the death of the animal and the commencement of the assay. The latter relates to the quality and robustness of the electrical recording and is particularly relevant to this data. Once an animal is sacrificed there is degradation of tissues and cells as the active processes that support membrane gradients start to fail. The longer the *time interval from death* the more chance of cell degradation. We provide the *intracellular electrophysiology stimulus* for each protocol and delineate whether a data item is measured or predicted with *measurement datum* and *predicted data item* as sub-groups. The datasets for protocol *electrical admittance dual-sine stimulus* describe the *electrical admittance* which is *measured* at both frequencies, and the membrane capacitance is *calculated* at both frequencies.

The 6^th^ group is a *transformed data set* where we divide the classes into sub-groups based upon which protocol was used (S4 Fig). For the protocol *electrical admittance dual-sine stimulus* there are three transformed data sets. The first is the *linear (voltage-independent) data set* sub-group with datasets: mean linear capacitance; mean membrane and series resistance, and the *voltage drop across the membrane*. The *non-linear (voltage-dependent) data set* is a sub-group with datasets: non-linear membrane capacitance, (NLC) (including the peak NLC value); the displacement charge (including the maximum displacement charge); the voltage sensitivity; and the potential of maximum sensitivity. The last sub-group is the data set of predicted values after fitting to the 2-state Boltzmann function. The values were determined upon fitting the capacitance versus membrane potential data to the capacitance form of the 2-state Boltzmann function [105] or upon fitting the charge versus membrane potential data to the displacement charge form of the 2-state Boltzmann function [106]. The electrical admittance dual-sine stimulus is an over-determined assay, and we provide an estimate of parameters at two frequencies and stipulate which estimate we report given the noise and electrical parameters of the cell [107–109]. We provide one sub-group for the protocol *membrane current*, *I as a function of membrane potential*, *V (I vs V) plot*. It contains three datasets the mean and standard deviation of the steady-state current (*I_mean);* the reciprocal of the conductance (*R*_*b*_) and the membrane potential. These four sub-groups of the transformed dataset contain key variables commonly reported in scientific communications.

The datasets, groups, and sub-groups have associated contact and context information [68, 72]. Each file reports the date when the experiment was conducted, the original cell number of the recording, the name of the researcher who conducted the experiment, the name of the person responsible for this data, the name of the data curator and the name of the funding source. Each group, sub-group and data file has a description that was either written by the authors or imported from existing ontologies. All datasets that are associated with standard units of measure have the additional *attribute* named *units* (e.g., units = *micrometer*, when describing length). Because HDF5 does not readily support Greek symbols like μm, or superscripts and subscripts, we write out the units in long-form; *e*.*g*., *micrometer* × *micrometer* (cf. μm^2^) when describing the area.

Concerning all datasets, our first attempt was to make them of type *compound* (which are identical to type *struct* found in C and MATLAB). This is a popular type, as it provides for related data collections of different sizes and types (e.g., string, integers, doubles) to be bundled together. However, we did not adopt the compound datatype, because HDF5 does not support adding metadata as attributes to the *fields* found within a compound datatype. Attributes are only associated with *objects* (e.g., groups and datasets) and a *field* in a compound datatype is not an object. Consequently, we decided to use datasets exclusively, while employing attributes to add descriptive mark-up. This is because our philosophy is to make use of extensive descriptions, and the use of the *compound* datatype would not only have made the mark-up of the metadata cumbersome, it could also lead to ambiguous interpretations of the metadata. This translation produced a large number of small datasets which can slow down retrieval. To help improve performance we store these small datasets as *compact* so that the data is stored with the metadata header. We did not bundle the data from different cells but described and translated them separately into named sensory files. We did this because bundling makes the write-up of metadata more cumbersome. We incorporated some conventions; all group and sub-group names were single words or phrases of the lower case unless they contained a well-known abbreviation (e.g., DC). In contrast, dataset names were contiguous words connected by an underscore. We did this because of the ease of re-reading the values back into MATLAB, where variable names must be labeled without white spaces.

### Packaging, licensing and storing the data

The package contains six main items: (1) the original unstructured data in MATLAB; (2) the data files translated to HDF5 with metadata; (3) MATLAB scripts that facilitate the translation of the data from MATLAB to HDF5; (4) MATLAB scripts that facilitate the translation of the data back to MATLAB from HDF5; (5) MATLAB scripts used to analyze the data; and (6) the OWL file describing the variant or application ontology. Data collections cannot usually have restrictions on their use and require a Creative Commons Zero license (CC0). We include the original data (item 1) as this is considered good data management practice since it allows the researchers to go back to the original file and to check for errors or inconsistencies. To promote the dissemination of the Data Management Plan we also impose no restriction on the use of the scripts and use the same Creative Commons Zero license. The MATLAB scripts can be found at the repositories. The OWL file was also deposited with the National Center for Biomedical Ontology. When the application ontology is revised, the new version will be uploaded to NCBO.

Best practices indicate that valuable data collections should be stored in multiple repositories in order to enhance availability and reduce the likelihood of data loss. The selection of a suitable repository is a challenge since there are only a few that specialize in this particular type of data. While the CARMEN portal and repository did not require rich descriptive metadata as generated here, it was probably the most suitable candidate; unfortunately, it is no longer available for data deposits. The IMPC [39] has tested the hearing of engineered mice with the non-invasive auditory brain stem methodology. This procedure and results are partially curated with the Mammalian Phenotype Ontology [98] where the raw and transformed data are available for download. However, IMPC does not provide a place for others to deposit their data. There is only one other repository compatible with the contents of our collection: Collaborative Research in Computational Neuroscience (CRCNS) [69], which specializes in neuroscience data (e.g., time series, imaging, and electrophysiology). We have established a sub-folder on the small discipline-specific repository CRCNS to house electrophysiological data collected from cells or anatomical structures that originate from the *inner ear*. In addition to CRCNS, we selected: Zenodo [110], and Digital Commons at the Texas Medical Center Library [111]. We choose TMC because of our affiliation, and Zenodo because there is no fee, it is not based in the U.S. (cf., CRCNS and TMC repository), and it is guaranteed to be maintained for 20 years.

Related to the repository selections, was the application of a Digital Object Identifier (DOI) to the data collection. Best practices stipulate that a digital object should have only one DOI associated with it. Since some repositories automatically assign a DOI, while others will accept a previously assigned DOI, we applied a strategy both in the repositories we submitted to, and in the order in which those submissions occurred. In order to facilitate a single DOI, we first submitted to CRCNS, a repository that will automatically assign a DOI to the data collection. Once the DOI was assigned, the data collection was submitted to Zenodo [110], and, because of our institutional affiliations, the TMC Digital Commons [111]. If the data collection needs revisions or additions, we will submit them in the same order. Updates will be uploaded to CRCNS and changes will be written in the text that describes the data collection. The same DOI is used and only if the versions are significantly different will both versions be stored. The changes will then be made to Zenodo and TMC.

## Results and discussion

### Comparison with other data formats

Our approach to develop a variant ontology to describe a data collection, and then use the ontology as the basis to design the data architecture within the hierarchical storage format HDF5, provides for a structurally simple data file that is intuitive and highly human-readable (Fig 1). Given that the necessary context is preserved by use of metadata (via the HDF5 attributes and the variant ontology), and this construct allows for aggregation using the hierarchal strengths inherent to HDF5, it should make it easier for researchers, whether they are familiar or unfamiliar with such data, to understand and reuse them for their own purposes. In addition, given the hierarchal nature of the HDF5 format, grafting the ontological structure onto the data collection as a framework seemed like the best approach. It provides a reliable, predictable structure for perusing the file with a computer program. In our opinion, this makes the data easier to understand at a glance, and easier to evaluate before the aggregation process is initiated. It also makes it easier to search the file with any HDF-capable editor in order to find a data point of interest before aggregation. If more complex data structures are required, this construct can be scaled-up by the expansion of the variant ontology. For example, if a researcher used a different protocol to interrogate the cell this protocol could be added to the application ontology with any associated new classes imported or defined. If a researcher interrogated a native outer hair cell isolated from a different rodent, the details could be imported and added to the application ontology. We made a relatively simple construct first to avoid a common pitfall of ontological development: the creation of a burdensome data structure [56].

This construct could also be readily expanded to describe other electrophysiology assays. Consider, the auditory brain stem response used to characterize the hearing of engineered mice by IMPC [39]. This *assay* is a planned *in vivo* electrophysiology process. The anesthetic agents would be described under the *chemical substance* class. Sound pressure level, (SPL) is a *quality* factor that would need a new class. The click and chirp sound stimulus would be described. The *measurement datum* is a voltage and encompasses the aggregated potentials at the brain regions (cf. membrane potential described for this assay). The *organism* is the *mouse* where the genotype, phenotype and comparative information (e.g., age, weight) would be included. The *anatomical entity* would describe the parts of the brain or nerve connections (e.g., cranial eighth nerve, cochlear nucleus) that are stimulated. The *device* class would describe the amplifiers, heating blanket, sound-booth and electrodes used. The *transformed data sets* would describe the data normally reported, including results from thresholding the waveforms, and a description of the various waves and the resulting hearing outcomes. In this way, this *in vivo* assay could be described with this expanded application ontology, translated with annotations to HDF5 to promote the interoperability and reusability of such commonly attained data collections.

Our approach differs significantly from BrainFORMATS [112] and NeuroData Without Borders (NWB) [70]. BrainFORMATS, while offering a powerful and flexible model for storing data, requires serialization of metadata values as JSON objects, often within attributes, providing a less direct structural path to those values and their meanings. In comparison to our format, which mirrors the structure of the ontology employed, BrainFORMATS separates data collections and at least some of the metadata into two silos at the top level [112]. As a format, it is well suited to describing index map relationships for purposes of connecting images to a series, for instance. It employs a well-designed Python module, with an associated Application Programming Interface (API) for interacting with the file. Python is particularly well suited for such data operations, being a flexible language with wide scientific support. However, given that our data was originally in the popular MATLAB format, this data collection did not need to make use of BrainFORMAT’s specific tools and did not require any additional complexity of structure, and hence we did not adopt BrainFORMAT.

Likewise, the authors investigated the NWB format [70], which provides an extremely effective system for serializing time-series data for aggregation, but which seemed to provide a less structured and intuitive platform for serializing our data. The upper-level file structure has a well-defined, predictable format suitable to the machine-aggregation that this modality is designed to easily provide. However, when looking over the format specification, we found that fitting the data collections for this project into the NWB format would have been difficult. Applying the descriptive metadata we proposed would have required the addition of structures beneath the NWB format upper levels, which would have necessitated additional scripting by anyone seeking to aggregate the data with other NWB files. This eliminates much of the advantage provided by the generically traversable NWB format. We decided to proceed with our ontology-based data architecture since it provided a structure that was easily human-readable, easy to traverse programmatically after minimal examination, provided a significant level of descriptive metadata, and required only scripts written in MATLAB in order to generate the HDF5.

Our format shares some commonality with the Allotrope Data Format (ADF). ADF was developed by OSTHUS and spearheaded by the Allotrope Foundation; a consortium of industrial companies and partners [113]. The framework is designed to standardize the acquisition, exchange, storage and access of analytical data, (e.g., mass spectrometry). Like our format, it makes use of ontologies (Allotrope Foundation Ontologies) and the HDF5 storage format, but in their case, the data structure is constrained by the shape constraint language (SHACL). SHACL (cf. OWL) is designed for expert producers and users of analytical data. Our format preserves inferred class relationships by transplanting them into the tree-based group structure of an HDF5 file. In contrast, the Allotrope Data Format uses the data cube mechanism to record some more complex relationships within HDF5, including axioms, that the OWL format is capable of encoding. However, the data cube format is not necessarily required to encode such relationships in HDF5 and does not provide a particularly human-readable way of doing so. As an alternative, the data architect has developed a method of using HDF5 attributes to encode deeper ontological relationships in a manner congruent with the data format presented in this paper. The result serves as an extension of the authors' data format; which is to say it is much more human-readable than axioms represented by a data cube, highly extensible, and can be navigated and parsed in exactly the same way as already described.

### Transformation to HDF5 from MATLAB and harvest from HDF5 by MATLAB

MATLAB has both a high and a low-level API for working with HDF5. Despite these capabilities, the overall documentation provided by Mathworks for transformation to HDF5 was limited and opaque. This is contrary to the level of documentation normally offered by Mathworks. Much of the information necessary to make the transformations from MATLAB to HDF5 was provided by The HDF Group and required a significant amount of trial and error on our part before we could craft satisfactory transformations. This part of the data management plan was time-consuming and would benefit from further development and documentation. This is contrary to the reverse-translation from HDF5 to MATLAB, which is straightforward and intuitive. Once the HDF5 file is open (using the *H5F*.*open* command) the use of the *h5info* command (e.g., INFO = h5info(‘*nameofile*’)) reveals the nested hierarchical structure of an HDF5 file within the variable named INFO. This structure is readily represented in MATLAB as a compound type (i.e., structure). For example, to evaluate the architecture below the Group (upper case nomenclature used by MATLAB) named *morphology* **(**Fig 2B**)**, one would first establish where this Group is within the file architecture retrieved within a script or at the command line:

DATA = INFO.Groups(3).Groups(1).Groups(2) as *morphology* is nested below Group named *cell* (1^st^ layer), and the sub-Group named *outer hair cell* (2^nd^ layer). The variable DATA would provide the contents of a structure with fields: *Name* of the *Group* (i.e., *morphology)* and the *Groups*, *Datasets*, *Datatypes*, *Links*, and *Attributes* (which are MATLAB key names) found below it. In this case, there are two Groups named: *measurement datum* and *predicted data item*. To explore the Group *measurement datum* further, and examine the properties of the *Datasets* stored below this directory structure, the label *Datasets(1)* or *Datasets(2)* or *Datasets(N)* (where *N* is a total number of datasets found within this sub-group) is added at the end of the query, e.g.,

DSET = INFO.Groups(3).Groups(1).Groups(2).Groups(1).Datasets(1).

Examination of variable DSET would now provide information on the first *Dataset*, including *Name ‘cell_diameter’*; the type of data stored found under *Datatype*; the space allocated for the data found under *Dataspace*; the metadata found under *Attribute*s.

In this way, it is straightforward to map the hierarchical arrangement of HDF5 to the nested structure format within MATLAB to retrieve the variables of interest for aggregation or to perform analysis with the tools provided within MATLAB. This illustrates that our simple construct provides a predictable structure for harvest.

### Data aggregation

Mechanical aggregation may pose some potential difficulties, in that, until other files appear in this format, customizing scripts to traverse the nodes of the file will be necessary. However, some of the same issues can be seen even in formats developed to ease data aggregation. Such designs may provide for consistency of some top-level folders, and structure for particular types of data collections (e.g., NWB, time-series data are the main focus), but often provide little guidance for creation and harvesting of other types of lower-level data and metadata with their tools. In our experience, programmatic aggregation of data across files is primarily a challenge when facing inconsistent (i.e., dirty) data, and inconsistent (heterogeneous) data formats. If the data is of reasonable consistency, as provided here, and if it is in a predictably traversable format, harvesting, and thereafter aggregating, the data, is simply a matter of traversing the relevant nodes of the file.

### Level of granularity

The process of converting, describing, and archiving this data collection raised a number of issues. One of the most subjective questions is how to determine the optimum level of granularity for the descriptive metadata applied to a data collection. There is not a rule here; others have elected for relatively minimal metadata, whereas we elected to apply a more in-depth layer of metadata to the data collection that, although more extensive, is actually not that much more than that established by the MINI guidelines [68, 71]. Our approach is similar to Zehl and colleagues who proposed the use of expansive metadata to ensure the steps and processes are reproducible and the data can be reused [114]. Research time required to acquire and organize metadata could be significantly reduced if the data structure was established before the commencement of such assays and we recommend this in the future. We also suggest that both scientists and architects collaborate to consider whether additional metadata (beyond the MINI) is needed to sufficiently describe the data to others removed from the original context of the experiment. We do note that a single ontology cannot describe the plethora of terms associated with this data collection (Table 1**)** and we suspect that this will be typical of other complex and heterogeneous data collections. OBI was chosen as it was developed to describe experimental investigations. Ontologies that were developed to describe computational based neuroscience studies (e.g., [95] and [102]) were less useful to describe this experimental data, as was the Ion Channel Electrophysiology ontology (ICEPO) [115], which described electrical and temporal properties of ion channels. In our case the voltage sensor [116] found within the lateral membrane of outer hair cells is not an ion-channel.

### Matching data collections to the most suitable repository

Many organizations (e.g., journals, government agencies, societies, and consortia) are providing guidelines for standards to adopt when sharing data [117] and making recommendations on the repository to use as they refine and accept these standards. A fundamental issue for the scientist is to locate the most appropriate place to deposit their data collections for future discovery and re-use. There are several useful resources for scientists to peruse when deciding which repository is most appropriate, including the German-based Registry of Research Data Repositories [118], and the UK-based fairsharing.org (FAIRsharing [119]). In some cases, it may be obvious to a researcher where their data should be deposited. For example, physiology data that describes measurements made on human subjects can be placed in the PhysioNet repository [120] which includes auditory-based measurements (e.g., evoked auditory responses). Data on hearing health of babies and children can be placed in the Children’s Hospital of Philadelphia Research Database [37]. Unfortunately, if similar measurements were made on small mammals like cats, guinea pigs, and rats there does not appear to be a vetted repository suitable to house these collections. There are non-vetted resources like CRCNS [69] and other repositories for electrophysiology time series data with emphasis on cardiovascular measurements (such data can be deposited with the Electrophysiology Data Discovery Index | The CardioVascular Research Grid [121]). There is also a Community (a term used by Zenodo.org) within Zenodo.org [110] that accepts data collections, and other products that describe electrophysiology and imaging data performed *in vivo*. It does not accept data collections or products of *in vitro* electrophysiological measurements as described here.

One example of the dearth of essential metadata in many curated data collections would be the results we discovered when we performed some searches in Dryad [122]. As of writing this article, we found in Dryad eighty-six [86] data collections when searching for "electrophysiology", eight [8] when searching for “voltage clamp”, and two [2] when the search item was “outer hair cell”. One of the two “outer hair cell” data collections contains experimental data similar to that discussed here but performed with cells isolated from *mus musculus* [123]. We note pertinent descriptive metadata, as espoused in MINI consortium documents [68, 71] was not provided (e.g., the sex, age and weight of the animal), nor were these metadata found in the published peer-reviewed journal article [124]. Clearly, the scientists creating such data collections need to appreciate the value of such reporting, which has been woefully undervalued by the scientific community, and the librarians and data architects that manage repositories need to find ways to ensure such MINI-compliant descriptive metadata is there to make these repositories more valuable.

To address the deficiency of suitable repositories, research communities could create subject-specific repositories. This would provide the researchers with more autonomy over the data collections they helped to create. However, this requires resources and expertise that are not always available. We note that if researchers continue to be forced to make use of the general repositories like Zenodo, Dryad, and Dataverse [125], then resources will still be needed later as they are mined to address new research questions. A compromise may be to set-up subject-specific repositories within these general repositories. In this way, researchers could piggy-back on their infrastructure, but have some control over the quality and type of data collections deposited. Zenodo allows this through its Community-based system as does Dataverse. This needs much more patronage by the entities who fund the research especially to support the establishment and maintenance of such virtual data libraries.

A related issue is how communities should manage such virtual libraries to ensure that deposited data collections adhere to some standard with respect to the data structure used and the quality of the data. The first problem we have discussed in detail and the incoming data could be checked for inconsistencies by suitable tools (e.g., OpenRefine [126]). Determining the data quality will require community members of the sub-field to validate data and data transformations, and this will require additional resources. We note that if there are aberrations within a data collection then they will be more likely to be found upon the aggregation of similar data collections [34]. Such events may be errors overlooked by the data producer or reveal new phenomenon only present in some of the data collections.

## Concluding remarks

In the past scientists have largely worked within "their world", and made use of their instruments (virtual or physical) to collect, compute and analyze their data. In the past, there was no urgency to develop intuitive, human-readable data structures that both the public and their scientific peers could readily understand. Indeed, in many cases, their data was in such an unstructured format that others would struggle to understand the contents. In many other cases, research data was lost as hard drives expired. The limits of this modality are articulated well by Freedman, Cockburn, and Simcoe [14]. We show that a poorly structured data collection can become human-readable through the use of extensive descriptions and by employing an ontology. The ontology satisfies the need for architecture or *schema* to provide overall structure, with allowable attributes and classes, and it also provides a controlled vocabulary to express durable and unambiguous definitions for the terms placed within the *schema*. We made use of HDF5 as it is a flexible storage format and permits marking-up the metadata to the digital objects [127]. Although others may debate the utility of the nested data structure we implemented, especially at the lower levels of architecture, we propose that transforming unstructured data into an ontology-based one with the structure of a defined HDF5 file is a viable strategy for the future. For example, this approach of compiling the directed root trees is a useful model to build work-flows. Conceptual maps can be used to draft and refine such work-flows, and once an application ontology is formulated the OWL file can be used for querying attributes.

The scientist can still work within their environments but can subsequently share their data with the public and their peers. However, a key component of this strategy will be the development of tools to more easily transform OWL files into the latticework of intricate and often vast frameworks that describe a researcher’s data structures. Efforts are being initiated to do this, including the creation and adoption of the Investigation-Study-Assay (ISA) framework [128] which has developed a suite of tools to describe an experimental investigation from initiation to publication. The consortia FAIRDOM [129] has developed similar tools that are geared to system biology modeling including RightField [130] that allows for the import of the classes and sub-classes of an ontology from an OWL file into the fields of an Excel spreadsheet. This permits the scientist to annotate their electronic notes and produce templates (with pre-defined nomenclature) that can then be used to populate the experimental parameters recorded during an assay. However, this tool does not yet permit the import of definitions of the classes which would be needed for improved human-understanding. Their spreadsheets conform to the application ontology Just Enough Results Model (JERM) [131]. This model was rationalized to encourage its adoption by scientists and is prudent given their reluctance to provide metadata in the past (e.g., [41]). We assert that we should train scientists on the basis of good knowledge representation and provide them more opportunities to partner with information professionals to facilitate high-quality data curation. They will then see the benefits of producing machine and human-understandable data collections that can be used and re-used by themselves, their peers and the public. This will require the development of tools that ease and eventually automate the process of applying rich, descriptive metadata to data collections for researchers. Small teams should be encouraged to participate [49] to develop a diverse range of options that cater to different frameworks. Finally, we are excited to write that *PubData* (i.e., PubMed for data) is coming. PubData will be a searchable portal for locating and downloading data collections and other research products that should further encourage curation. The difference is the *journal* that describes the papers in *PubMed* is now replaced by a *repository* that describes and houses the data collections. This search engine, coined *DataMED* [132], is being developed by the bioCaddie (biomedical and healthcare data discovery index ecosystem) team [133] who are formulating the rules that will ensure data collections are *Findable*, increasing the probability that data collections will be reused.

## Data and scripts

The data, scripts and OWL file can be found at: (1) CRCNS repository http://dx.doi.org/10.6080/K0571975 with a direct link http://crcns.org/data-sets/ear/ear-1; (2) Zenodo https://zenodo.org/record/2818546#.XNrlJtNKhhE and at (3) Digital Commons at the Texas Medical Center Library https://digitalcommons.library.tmc.edu/baylor_datasets/1/. The OWL file can be found at https://bioportal.bioontology.org/ontologies/OBI_IEE.

## Supporting information

S1 Fig (A) Directed root tree for the *anatomical* arm showing the classes that describe the data.**(**B) Data architecture implemented to describe this arm with other main groups shown. The classes that were transformed into sub-groups, datasets, and data values are denoted by green, aqua and yellow.
(PDF)

S2 Fig (A) Directed root tree for the d*evice* arm showing the classes that describe the data. (B) Data architecture implemented to describe this arm with other main groups shown. The classes that were transformed into sub-groups, and datasets are denoted by pink and aqua.
(PDF)

S3 Fig Directed root tree for the *transformed data set* arm showing the classes that describe the data for both protocols. (PDF)

S4 Fig Data architecture implemented to describe the *transformed data set*. The classes that were translated to sub-groups and datasets are denoted by light purple and aqua.
(PDF)
