# Supplementary material for: Scientist and data architect collaborate to curate and archive an inner ear electrophysiology data collection
Source: PLoS One. 2019 Oct 18;14(10):e0223984. doi: 10.1371/journal.pone.0223984 (PMC6799921; doi:10.1371/journal.pone.0223984)
Supplement: S1 Fig — (A) Directed root tree for the anatomical arm showing the classes that describe the data.(B) Data architecture implemented to describe this arm with other main groups shown. The classes that were transformed into sub-groups, datasets, and data values are denoted by green, aqua and yellow. (PDF) [file pone.0223984.s001.pdf]

A

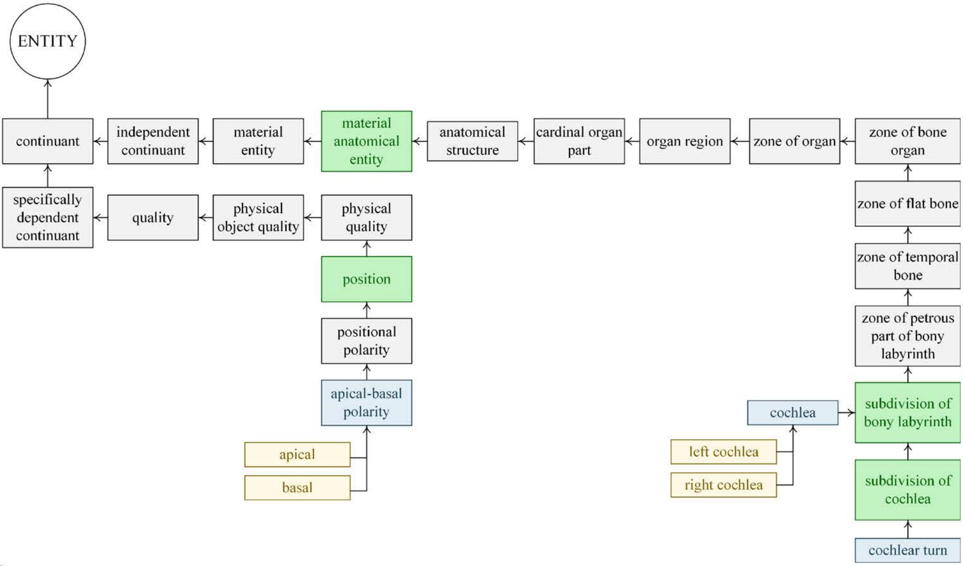

B

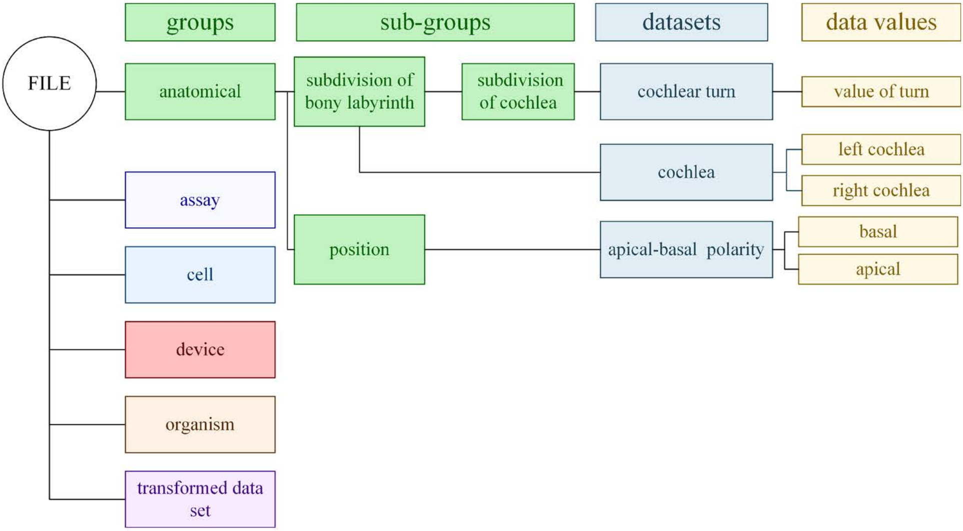

S1 Fig. **(A)** Directed root tree for the *anatomical* arm showing the *classes* that describe the data. **(B)** Data architecture implemented with other main *groups* shown. Classes that were transformed to sub-groups, datasets and data values are denoted by green, aqua and yellow.
