# Supplementary material for: Scientist and data architect collaborate to curate and archive an inner ear electrophysiology data collection
Source: PLoS One. 2019 Oct 18;14(10):e0223984. doi: 10.1371/journal.pone.0223984 (PMC6799921; doi:10.1371/journal.pone.0223984)
Supplement: S2 Fig — (A) Directed root tree for the device arm showing the classes that describe the data. (B) Data architecture implemented to describe this arm with other main groups shown. The classes that were transformed into sub-groups, and datasets are denoted by pink and aqua. (PDF) [file pone.0223984.s002.pdf]

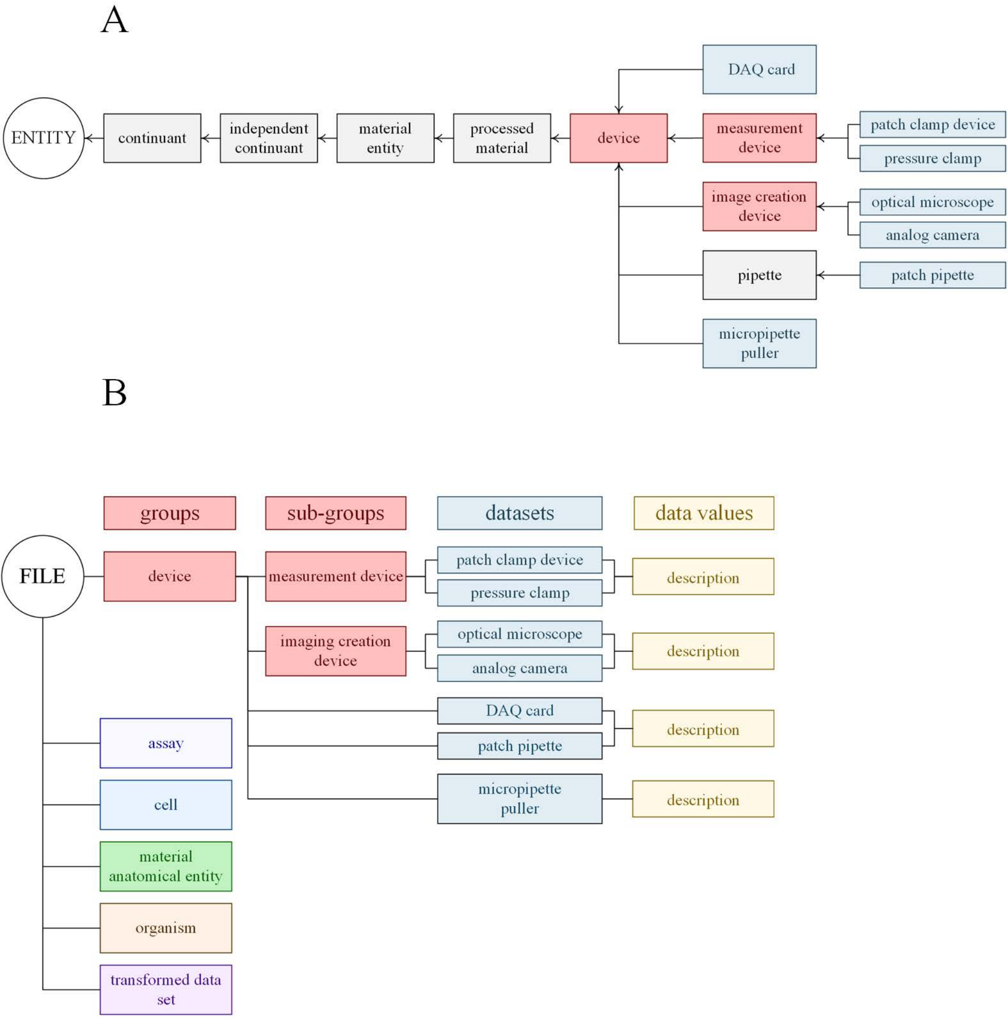


**S2 Fig.** (A) Directed root tree for the *device* arm showing the classes that describe the data. (B) Data architecture implemented to describe this arm with other main groups shown. The classes that were transformed into sub-groups, and datasets are denoted by pink and aqua.
