# Supplementary material for: Scientist and data architect collaborate to curate and archive an inner ear electrophysiology data collection
Source: PLoS One. 2019 Oct 18;14(10):e0223984. doi: 10.1371/journal.pone.0223984 (PMC6799921; doi:10.1371/journal.pone.0223984)
Supplement: S3 Fig — (PDF) [file pone.0223984.s003.pdf]

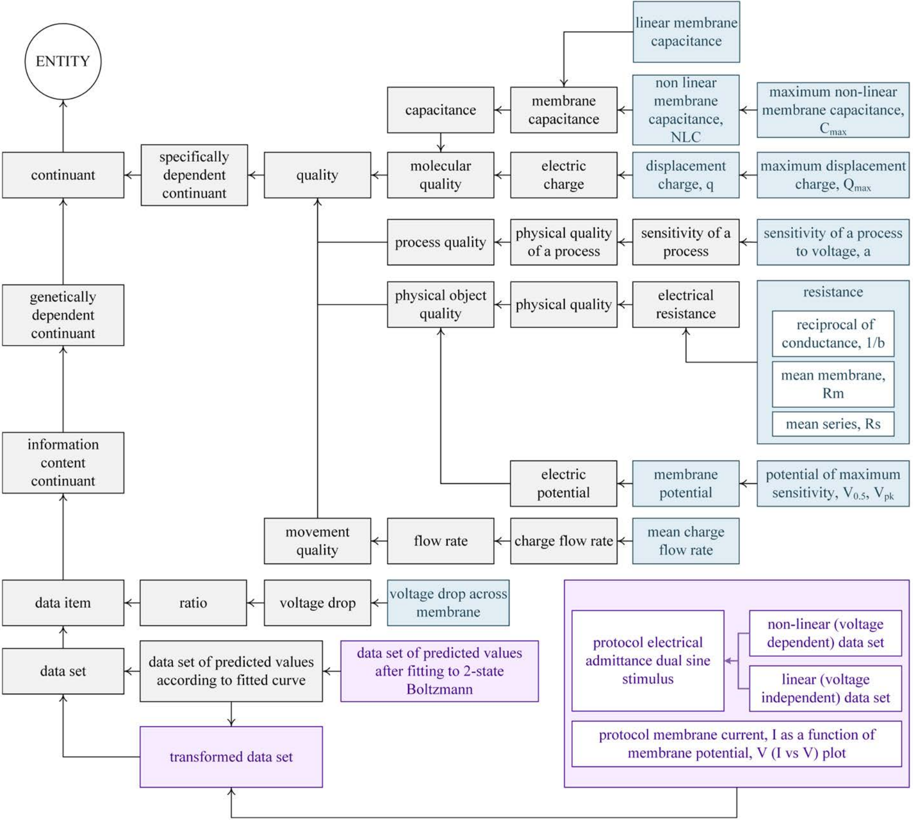

S3 Fig. Directed root tree for the *transformed data set* arm showing the classes that describe the data for both protocols.
