# Supplementary material for: Scientist and data architect collaborate to curate and archive an inner ear electrophysiology data collection
Source: PLoS One. 2019 Oct 18;14(10):e0223984. doi: 10.1371/journal.pone.0223984 (PMC6799921; doi:10.1371/journal.pone.0223984)
Supplement: S4 Fig — The classes that were translated to sub-groups and datasets are denoted by light purple and aqua. (PDF) [file pone.0223984.s004.pdf]

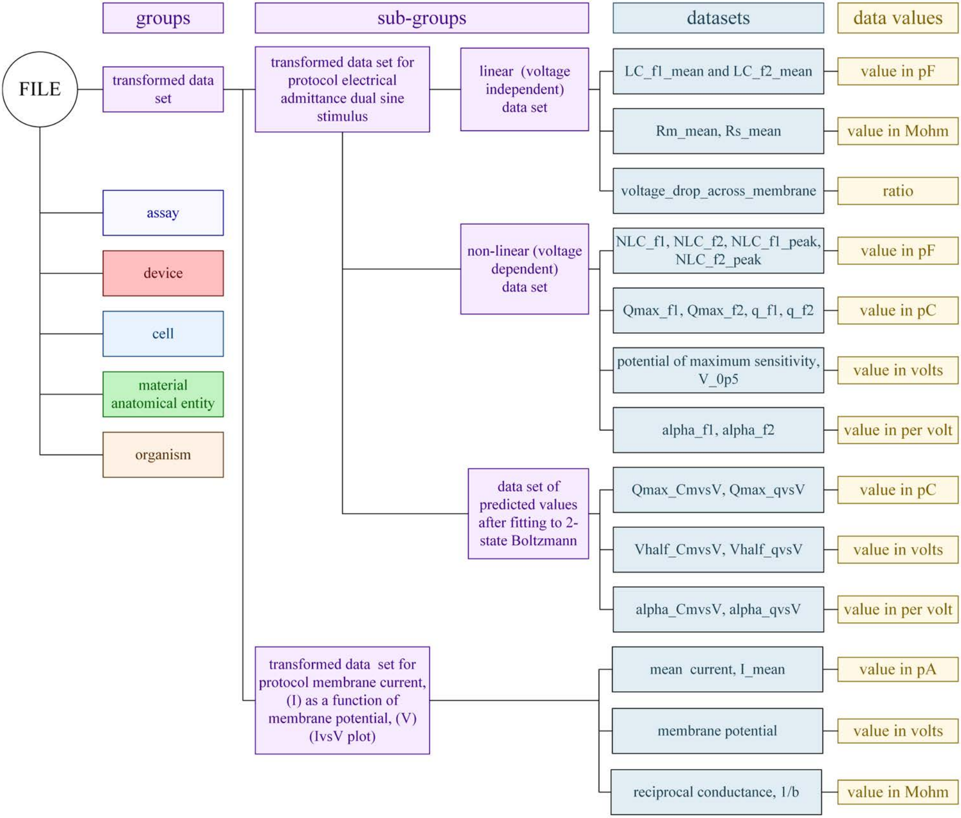


**S4 Fig.** Data architecture implemented to describe the *transformed data set*. The classes that were translated to sub-groups and datasets are denoted by light purple and aqua.
